# Ninth International Medical Congress

**Published:** 1887-08

**Authors:** 


### EDITORIAL.

### NINTH INTERNATIONAL MEDICAL CONGRESS.

The time is now near when the next International Medical Congress—the ninth triennial—will convene in Washington. As this will be the first one of them to meet in America, it is gratifying to find that the indications at present are that it will be a very successful one. The announcements already made of the scientific work to be done show no lack of interest, on the part of the medical world, in the object for which the congress meets. Many of the names announced include in their number men of such standing in the profession of medicine as are a guarantee that much of the work will be of a high scientific character. From a numerical standpoint, the registry-list to this date indicates that the attendance will be of the largest of any congress yet held, and embraces the names of several hundred Europeans, as well as of those of other foreign countries. The local committee of arrangements has been untiring in an effort to have the social feature of it in keeping with the greatness of the occasion, although the time of the year at which the congress will be held makes it an unfavorable one, socially, in Washington. The programme, as announced, embraces in addition to the social entertainments in the city, an excursion to Mount Vernon, and one, for the foreign members to Niagara Falls, with an escort of some of the committeee of arrangements.

Arrangements have been made with the principal ocean steamship companies for reduction of rates for those who will attend the congress, as well as for members of their families who may accompany them. A similar reduction has been made by the most important railroads of this country, and special excursion rates for a round-trip across the Continent for those who may wish to visit the Pacific Coast, after the adjournment of the congress. A committee of reception has been organized in each of the cities of New York, Philadelphia, Boston and Baltimore to aid foreign members of the congress, on their arrival, in their efforts to obtain requisite information regarding matters connected with the congress. In New York, the Hoffman House has been selected as the headquarters of the reception committee, and a Bureau of Information organized, under the supervision of members of that committee.

Careful effort has been made by all officials connected in any way with the congress to anticipate, and to provide, as far as practicable, for the needs of all of the large numbers who are expected to attend the great congress.

It will doubtless be the greatest event for the medical men of America that this country has seen, and all will hope that nothing may occur to mar the occasion to which so many of the medical world are now looking forward with so much hope and such pleasant anticipation.

